# Molecular Basis of AmpC β-Lactamase Induction by Avibactam in *Pseudomonas aeruginosa*: PBP Occupancy, Live Cell Binding Dynamics and Impact on Resistant Clinical Isolates Harboring PDC-X Variants

**DOI:** 10.3390/ijms22063051

**Published:** 2021-03-17

**Authors:** Silvia López-Argüello, María Montaner, Antonio Oliver, Bartolome Moya

**Affiliations:** Servicio de Microbiología and Unidad de Investigación, Hospital Son Espases, Instituto de Investigación Sanitaria Illes Balears (IdISBa), 07120 Palma de Mallorca, Spain; silviadaiana.lopez@ssib.es (S.L.-A.); mariadeldulcenombre.montaner@ssib.es (M.M.); antonio.oliver@ssib.es (A.O.)

**Keywords:** penicillin-binding proteins, PBP, avibactam, AmpC induction, PDC, β-lactam resistance, ST175

## Abstract

Avibactam belongs to the new class of diazabicyclooctane β-lactamase inhibitors. Its inhibitory spectrum includes class A, C and D enzymes, including *P. aeruginosa* AmpC. Nonetheless, recent reports have revealed strain-dependent avibactam AmpC induction. In the present work, we wanted to assess the mechanistic basis underlying AmpC induction and determine if derepressed PDC-X mutated enzymes from ceftazidime/avibactam-resistant clinical isolates were further inducible. We determined avibactam concentrations that half-maximally inhibited (IC_50_) bocillin FL binding. Inducer β-lactams were also studied as comparators. Live cells’ time-course penicillin-binding proteins (PBPs) occupancy of avibactam was studied. To assess the *ampC* induction capacity of avibactam and comparators, qRT-PCR was performed in wild-type PAO1, PBP4, triple PBP4, 5/6 and 7 knockout derivatives and two ceftazidime/avibactam-susceptible/resistant XDR clinical isolates belonging to the epidemic high-risk clone ST175. PBP4 inhibition was observed for avibactam and β-lactam comparators. Induction capacity was consistently correlated with PBP4 binding affinity. Outer membrane permeability-limited PBP4 binding was observed in the live cells’ assay. As expected, imipenem and cefoxitin showed strong induction in PAO1, especially for carbapenem; avibactam induction was conversely weaker. Overall, the inducer effect was less remarkable in *ampC*-derepressed mutants and nonetheless absent upon avibactam exposure in the clinical isolates harboring mutated AmpC variants and their parental strains.

## 1. Introduction

The worldwide emergence of multidrug-resistant Gram-negative *Pseudomonas aeruginosa* isolates has caused a substantial public health problem, which is exacerbated by few therapeutic options remaining [1]. Owing to their extensively proven efficacy and safety, β-lactams have been the drug of choice to treat infections caused by *P. aeruginosa* [2]. All β-lactams bind to and inactivate multiple penicillin-binding proteins (PBPs) with different affinities. PBPs play different roles in peptidoglycan biosynthesis. Roughly, high-molecular-weight PBPs catalyze peptidoglycan polymerization and the cross-linking of glycan strands (transglycosylase (*ponA* and *mrcB*; PBP1a and 1b) and DD-transpeptidase (PBP1a, 1b, 2 and 3)), cell wall elongation (*pbpA*; PBP2) and septum formation (*fstI*; PBP3). Low-molecular-weight PBPs catalyze DD-endopeptidase (*dac* and *pbpG*; PBP4 and 7) and DD-carboxypeptidase (*dacC*; PBP5/6) activities, necessary for the correct peptidoglycan incorporation and cell growth. The bactericidal activity of β-lactams requires the saturation of different PBP receptors, causing the inhibition of the aforementioned biological processes. However, intrinsic and acquired β-lactam resistance is one of the striking features of this microorganism. The chromosomal cephalosporinase AmpC (or *Pseudomonas*-derived cephalosporinase 1 (PDC-1)) represents one of the most menacing weapons among the extensive *P. aeruginosa* enzymatic warfare [3]. AmpC is an inducible broad-spectrum Ambler class C β-lactamase capable of hydrolyzing most β-lactams, excluding carbapenems.

Inducible serine AmpC β-lactamases are a common resistance mechanism among many Enterobacterales (*Enterobacter cloacae*, *Serratia marcescens* and *Citrobacter freundii*) and P. aeruginosa isolates. The induction phenomenon occurs via transient *ampC* overexpression due to the resulting accumulation of the activator muropeptide anhNAM-P5 (1,6-anhydro-*N*-acetylmuramyl-pentapeptides) after peptidoglycan metabolism disruption. The fact that the same muropeptide has been found to be the underlying cause of AmpC constitutive hyperexpression in *dacB* (penicillin-binding protein 4 (PBP4)) mutants, endorses PBP4’s key role during the induction process [4,5,6]. Antipseudomonal penicillins (piperacillin) and cephalosporins (ceftazidime or ceftolozane) are active against *P. aeruginosa* because they are very weak inducers of this chromosomal β-lactamase.

In the face of the continuous upscaling of β-lactamase-driven multidrug resistance (MDR) in Gram-negatives, new β-lactam-β-lactamase inhibitor (BLI) treatment options have become available. However, AmpC inhibition is out of the spectrum of most BLIs. Novel combinations such as ceftazidime/avibactam, ceftolozane/tazobactam, meropenem/vaborbactam and cefepime/zidebactam have based their activity against *P. aeruginosa* upon different strategies [7,8]. The avibactam β-lactamase inhibitor spectrum includes class C enzymes, which protect the partner β-lactam from AmpC-related hydrolysis [9]. While tazobactam does not have a major impact on the activity of ceftolozane against *P. aeruginosa*, the latter retains its activity against extremely high *ampC* (PDC-1) expression in addition to its improved PBP-binding profile [10,11]. Zidebactam, in spite of its fair inhibition capability towards class C β-lactamases, focuses its activity on β-lactam-enhancer properties derived from targeting PBP2 [12,13]. Finally, vaborbactam, a cyclic boronic-based narrow-spectrum BLI, does not offer additional protection against AmpC hyperproduction; however, it seems to enhance meropenem activity against *P. aeruginosa* [7].

Unfortunately, clinical isolates of *P. aeruginosa* resistant to ceftolozane/tazobactam (TOL/TAZ) and ceftazidime/avibactam (CAZ/AVI), expressing mutant variants of AmpC (i.e., PDC-221, -222, -223 and -322), have already been reported [14,15,16]. The studied AmpC mutants showed structural mutations in the omega (Ω) loop or adjacent residues. Such mutations appear to exert a concurrent effect on the catalytic properties of AmpC, reducing its susceptibility towards avibactam inhibition and enhancing its catalytic efficiencies towards ceftolozane and ceftazidime hydrolysis [17].

Furthermore, clinically relevant BLIs such as clavulanic acid, tazobactam and sulbactam have been shown to induce *ampC* expression at clinically relevant concentrations [18,19]. Livermore et al. described a similar effect for the non-β-lactam BLI avibactam, showing significant induction at higher than clinically relevant concentrations (32 µg/mL) and profound strain-to-strain variability. However, according to their work, such induction capacity would become clinically significant only if two conditions were met for the AmpC enzyme: loss of inhibition by avibactam while retaining inducibility [20].

Under these circumstances, knowing whether a novel BLI would be able to induce AmpC expression in avibactam-insensitive fifth-generation cephalosporin-resistant strains would be of great interest. On that premise, we examined the basis for the underlying AmpC induction in the presence of clinically relevant avibactam concentrations (≈50% of *C*_max_ after 500 mg standard dosage) [21] and the behavior of mutated AmpC enzymes (PDC-221 and -223) [15] upon induction in CAZ/AVI-resistant *P. aeruginosa* clinical isolates.

## 2. Results and Discussion

### 2.1. Molecular Basis of Avibactam AmpC Induction

**Figure 1:**
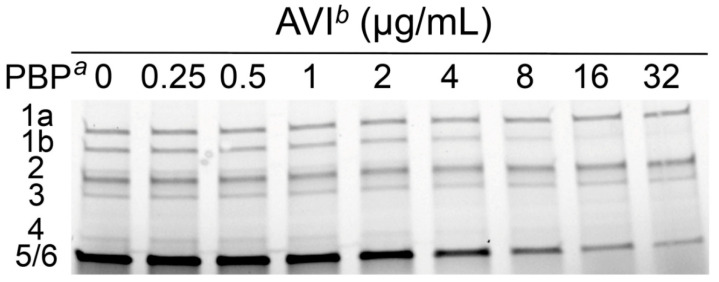
Illustrative example of a PBP-binding IC_50_ SDS–polyacrylamide gel. *P. aeruginosa* PAO1 cultures were grown to the midexponential phase (7.6 log_10_ CFU/mL), and membranes were isolated by ultracentrifugation. Isolated PBP-containing membrane preparations (0.5 mg/mL) were incubated in the presence of increasing concentrations of avibactam and afterwards were labeled with 25 µM bocillin FL. Labeled PBPs were separated by SDS-PAGE and detected using a Fluorimager. *^a^* Penicillin-binding proteins identified in *P. aeruginosa* PAO1. *^b^* AVI, avibactam, range of concentrations tested: 0.25–32 µg/mL.

To investigate whether the induction of *ampC* expression in the wild-type *P. aeruginosa* PAO1 strain was correlated to PBP4 inhibition, the fluorogenic bocillin FL PBP-binding assay was used. The PBP-binding IC_50_ values were determined by incubating PBP-containing membrane preparations in growing concentrations (0.25–32 µg/mL) of avibactam and two typical PBP4-binding AmpC inducers (cefoxitin and imipenem) and afterwards labeling with bocillin FL to determine the β-lactam concentrations that half-maximally inhibited (IC_50_) bocillin FL binding (Table 1). Cefoxitin and imipenem, as previously described, showed low PBP4 IC_50_ values, the lowest observed for imipenem (0.1 and 1.5 µg/mL, respectively) [11,22]. Imipenem bound to all PBPs with the highest efficiency with concentrations that ranged from 0.05 to 0.3 µg/mL, whereas cefoxitin binding values for non-PBP4 enzymes were >250-fold higher (range: 7.8–14.6 µg/mL), and no PBP1b binding was detected. Avibactam bound to PBP1b, 2, 4 and 5/6 (IC_50_ = 3.6, 4.2, 3.1 and 2.2 µg/mL, respectively). However, in contrast to previous works, no PBP1a and 3 binding was detected under our experimental conditions in the studied strain (Figure 1) [23]. Besides showing greater PBP4 affinity than previously reported values, avibactam effective concentration was the highest observed from all the three compounds tested (3.1 µg/mL) [23]. Consequently, the differential PBP affinities could be attributed to strain-to-strain variability [20].

### 2.2. Avibactam Target Binding in Live P. aeruginosa Cells

**Figure 2:**
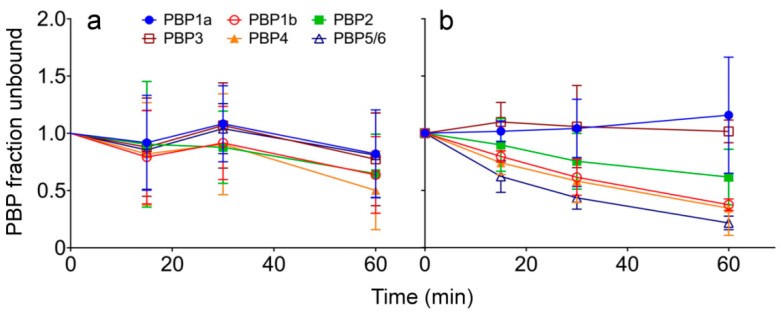
Time-course of intact cells and isolated PBP-binding assay. *P. aeruginosa* PAO1 cultures (**a**) and isolated PBP-containing membrane preparations (**b**) were incubated for 15, 30 and 60 min in the presence of 4 µg/mL avibactam (AVI). After isolating PBP-containing membranes, preparations were labeled with 25 µM bocillin FL. The graphs represent the PBP fraction unbound relative to each time control over time. The mean PBP fraction unbound values from at least three independent experiments ± standard deviations are shown.

To gather further information regarding the PBP4-related *ampC* induction by avibactam, the time-course of PBP-binding in live *P. aeruginosa* cells was determined as previously described with several modifications [24]. We used intact PAO1 cells in the late exponential growth phase with an initial inoculum of ≈4.0 × 10^7^ CFU/mL. To infer the outer membrane permeability-limited target binding, a matching experiment with isolated PBP-containing membranes (from previously lysed cells) was similarly performed in the presence of 4 µg/mL avibactam (AVI) (fixed concentration used for minimum inhibitory concentrations and pharmacokinetic model studies). Figure 2 shows the intact cells’ (Figure 2a) and isolated membranes’ (Figure 2b) binding affinities. No significant PBP1a, 3 and 5/6 binding was observed for the whole-cell assay, whereas 20% of PBP1b and 2 fractions and up to 50% of PBP4 were bound by avibactam after 60 min of incubation. Previously reported data showed that avibactam uptake was indeed limited by the outer membrane permeability barrier, and its activity could be further enhanced upon permeabilization in Gram-negatives, including *P. aeruginosa* [25]. Unsurprisingly, isolated membranes assay determined a more extensive inhibition of all the PBPs but PBP1a and 3, binding up to 68 and 82% of PBP4 and 5/6 total fractions respectively. As previously suggested by other authors, we confirmed PBP4 binding by avibactam in *P. aeruginosa,* in both whole cells and isolated membranes [20,26].

### 2.3. qRT-PCR-Based ampC Induction Assays

To investigate whether the binding of avibactam to PBP4 in live cells was correlated with *ampC* induction, qRT-PCR was performed to determine the time-course expression of β-lactamase mRNA (AVI 16 µg/mL; t = 0, 30 and 180 min). Typical AmpC inducers, imipenem and cefoxitin, were used as comparators (IPM 8 µg/mL, FOX 64 µg/mL). To assess the potential clinical implications of *ampC* induction in the clinical worst-case scenario, two clinical isolates belonging to the high-risk clone ST175, harboring AmpC structural modifications in the Ω loop or adjacent residues and their parental wild-type AmpC isolates, were also studied. Described AmpC mutations were previously shown to reduce susceptibility to avibactam inhibition and enhance ceftolozane and ceftazidime catalytic efficiencies simultaneously [15,17].

Table 2 shows the basal *ampC* mRNA expression levels for each of the strains studied relative to wild-type strain PAO1. As previously demonstrated by our group and other independent researchers, PBP4 inactivation (PAΔ*dacB*) elicited significant *ampC* hyperexpression; the highest relative expression values were observed at the 180 min determination (1100 ± 243). Both initial ST175 isolates, 101-E5 and 109-E9 (wild-type AmpC), and their CAZ/AVI post-therapy resistant isolates, 103-H8 and 110-G8, showed an even more extensive *ampC* relative expression (2400 ± 1100, 1700 ± 382, 5400 ± 2900 and 3500 ± 1600, respectively) consistent with the *ampR*-G154R-resulting enhanced *ampC* expression observed in nearly all of the ST175 isolates [15,27,28]. Inactivation of the three low-molecular-weight (LMW) PBPs (PBP4, 5/6 and 7) in PAΔ*dacBdacCpbpG* elicited the most remarkable cephalosporinase constitutive overexpression (58,000 ± 26,000) after 180 min.

**Figure 3:**
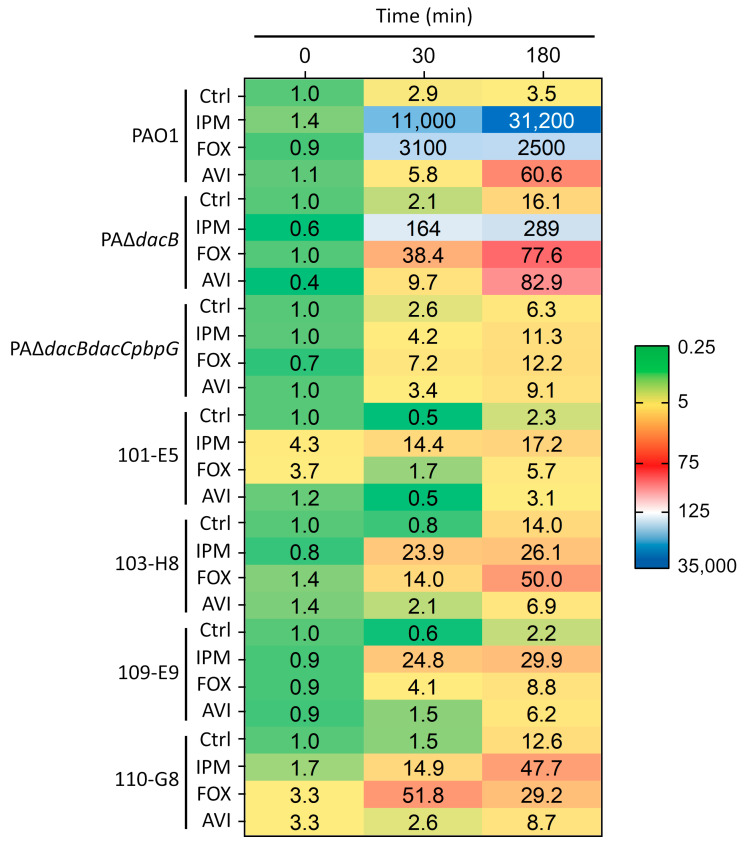
Relative *ampC* qRT-PCR expression under basal conditions (Ctrl) and after induction with 8 µg/mL imipenem (IPM), 64 µg/mL cefoxitin (FOX) and 16 µg/mL avibactam (AVI) in strains PAO1, PAΔ*dacB* (PBP4 knockout mutant), PAΔ*dacBdacCpbpG* (PBP4, 5/6 and 7 triple knockout mutant), 101-E5 and 109-E9 (OprD Q142X, AmpR G154R) CAZ/AVI sensitive ST175 high-risk clone clinical isolates and 103-H8 (AmpC E247K; PDC-221) and 110-G8 (AmpC DelG229–E247; PDC-223) CAZ/AVI-resistant ST175 high-risk clone clinical isolates. Differentially expressed *ampC* levels at times 0, 30 and 180 min relative to each strain basal initial determination (Ctrl; time = 0 min) are shown. Results are averages of at least two sets (biological replicates) of two technical replicates.

The AmpC mRNA levels under basal conditions and after incubation with 8 µg/mL imipenem, 64 µg/mL cefoxitin and 16 µg/mL avibactam for 0, 30 and 180 min in strains PAO1, PAΔ*dacB*, PAΔ*dacBdacCpbpG*, 101-E5 and 109-E9 (OprD Q142X, AmpR G154R) CAZ/AVI sensitive and their 103-H8 (PDC-221; AmpC E247K) and 110-G8 (PDC-223; AmpC DelG229-E247) isogenic CAZ/AVI-resistant isolates are shown in Figure 3. The values represent each strain *ampC* relative expression normalized to its expression under basal conditions at t = 0 min. The most notable cephalosporinase induction was observed for the wild-type strain PAO1 upon imipenem incubation. The maximum values (31,200 ± 17,000) were observed by the end of the assay (180 min). As previously reported in works from our group and others, cefoxitin induction was less significant compared to imipenem, achieving maximum expression after 30 min incubation [11,20]. On the other hand, avibactam showed the lowest *ampC* induction capacity, however still significant (according to the >10 times threshold proposed by several authors) after 180 min incubation (60.6 ± 25.2) [20,29]. As opposed to observations in other species, avibactam is capable of inducing *P. aeruginosa* chromosomal cephalosporinase AmpC [20,26], although not as strongly as classical inducers such as imipenem or cefoxitin. Experimental induction capacity was consistently correlated with observed PBP4 inhibition values for each of the studied drugs (Table 1).

According to the premise that higher basal *ampC* expression levels (constitutive overexpression) determine lesser inducible endpoints, the PBP4 (*dacB*) mutant showed a much lower *ampC* inducibility compared to its parental strain PAO1 for both imipenem and cefoxitin for all the observations, 107- and 32-fold lower (289 ± 138 and 77.6 ± 35.7) for the knockout strain at 180 min, respectively [6]. Surprisingly, avibactam caused a slightly higher *ampC* expression increase in *dacB* mutant, especially towards the end of the assay (180 min). This observation could be attributed to the concomitant inhibition of LMW PBP5/6 and 7 (*dacC*, *pbpG*) beyond PBP4 inactivation [4,22]. In fact, regardless of a remarkably higher relative basal *ampC* expression compared to PAO1 (58,000 ± 26,000; t = 180 min), triple PAΔ*dacBdacCpbpG* mutant showed no significant avibactam induction (9.1 ± 4.3) (Figure 3).

In addition to the lower induction capacity thoroughly observed for the AmpC-derepressed mutants, the lower imipenem β-lactamase induction effect in ST175 clinical isolates (101-E5, 109-E9, 103-H8 and 110-G8) could be further explained by the observed OprD mutations [15]; that is, restricting imipenem periplasmic uptake would reduce the number of molecules capable of binding to the low-molecular-weight PBPs, which, in turn, cause the β-lactamase expression induction [4,22]. Moreover, AmpC structural mutations have been shown to reduce imipenem resistance and presumably enzyme’s hydrolytic capacity towards this compound [30].

Regarding cefoxitin, all the ST175 clinical isolates showed a significantly diminished induction, virtually negligible in the wild-type AmpC carriers 101-E5 and 109-E9 (5.7 ± 2.8 and 8.8 ± 7.9, respectively), but still within positive induction threshold values at 180 min in the 103-H8 and 110-G8 resistant isolates (50 ± 36.5 and 29.2 ± 13.1, respectively); higher after 30 min incubation in the 110-G8 isolate (51.8 ± 13.2). This observation could be intimately linked to its otherwise *ampR* mutation-mediated high-level *ampC* derepression. Conversely, induction caused by clinically relevant avibactam exposure (16 µg/mL) [21] was not significant for any of the ST175 clinical isolates (3.1, 6.9, 6.2 and 8.7 at 180 min); indeed, mRNA expression was consistently lower than the observed for the control arms in the 103-H8 and 110-G8 CAZ/AVI-resistant mutants (14 and 12.6, respectively). Hence, the observed loss of inducibility appears to be linked to the transcriptional regulator AmpR mutation (G154R) [28].

In summary, the results presented in this work are in accordance with previous studies suggesting a link between avibactam *ampC* induction and PBP4 inhibition. Furthermore, we demonstrated “in vivo” PBP4-derived induction process and outer membrane permeability-limited avibactam target site penetration and binding. This may explain the added synergistic effects of CAZ/AVI when coadministered with an outer membrane permeabilizer [25]. What is more important is that, even when the foretold conditions for the clinical significance of avibactam induction are met (i.e., mutated AmpC with loss of affinity for avibactam while retaining inducibility) [20], *ampC* variants studied in this work (PDC-221 and -223) were not reactive to avibactam induction. In terms of bacterial physiology and fitness, it seems reasonable not to retain full inducibility once the derepressed enzyme expression has already been selected [6]. Understanding the dynamics of mutational *ampC* regulation and resistance to antimicrobials and β-lactamase inhibitors will definitely help the rational development of novel compounds and combinations that are less prone to select mutational resistance.

## 3. Methods

### 3.1. Bacterial Strains and Antibiotics

The wild-type reference strain *P. aeruginosa* PAO1 [31] was used for PBP-binding, IC_50_ determination and quantification of the AmpC induction assay. *P. aeruginosa* knockout strains PAΔ*dacB* (PBP4) [6] and PAΔ*dacBdacCpbpG* (PBP4, 5/6 and 7) [22], and two previously characterized [15] pairs of TOL/TAZ susceptible/resistant XDR clinical isolates belonging to the epidemic high-risk clone ST175, were also used for the quantification of AmpC induction.

The isogenic clinical isolates pairs were obtained from two patients treated with TOL/TAZ. Susceptible isolates 101-E5 and 109-E9 (OprD Q142X, AmpR G154R) were obtained before the therapy onset, and the resistant isolates 103-H8 and 110-G8 were obtained during or after the completion of therapy. Resistance development to TOL/TAZ was linked to additional AmpC structural modifications associated with mutations in the Ω loop or adjacent residues in 103-H8 (AmpC E247K; PDC-221) and 110-G8 (AmpC DelG229-E247; PDC-223) isolates. Mutant forms of AmpC rendered the isolates resistant to CAZ/AVI as well [15].

Imipenem (IPM) was obtained from Fresenius Kabi, Barcelona, Spain, cefoxitin (FOX) from Laboratorios Normon, Madrid, Spain and avibactam (AVI) from MedChem Express, Stockholm, Sweden.

### 3.2. Intact Cells’ Time-Course of PBP-Binding Assay

*P. aeruginosa* PAO1 cultures grown to the midexponential phase (7.6 log_10_ CFU/mL) were preincubated in cation-adjusted Mueller–Hinton broth (CAMHB) at 37 °C (180 rpm) for 20 min. Afterwards, 4  µg/mL of avibactam (AVI) was added, and the bacterial cultures were incubated (37 °C, 180 rpm). Control and treatment samples were taken after 15, 30 and 60 min incubation, kept in ice and centrifuged (3220× *g* for 10 min at 4 °C). The bacterial pellets were washed in 30 mL phosphate-buffered saline (PBS) (pH 7.5) four times, resuspended in 10 mL of PBS and sonicated by using a Digital Sonifier Unit model S-450D (Branson Ultrasonics Corp., Danbury, CT, USA) at 40% amplitude for three 2 min bursts (while immersed in an ice bath) and centrifuged at 3220× *g* for 15 min at 4 °C. Membranes containing the PBPs were isolated by ultracentrifugation at 150,000× *g* for 30 min at 4 °C using an Optima L100XP Ultra centrifuge (Beckman Coulter, Inc., Palo Alto, CA, USA) and were resuspended in 60 μL of PBS. Total protein content was measured using the Quick Start Bradford protein assay kit with bovine serum albumin as a standard (Bio-Rad Laboratories, Hercules, CA, USA), according to the manufacturer’s instructions. To determine the PBP fraction unbound, membranes containing PBPs (20 μL, at 0.5 mg/mL) were labeled with a 25 μM concentration of the fluorescent penicillin bocillin FL [32]. Labeled PBPs were denatured and separated through 4–15% SDS–polyacrylamide gel electrophoresis (Bio-Rad Laboratories, Hercules, CA, USA). Labeled PBPs were visualized using a Typhoon FLA 9500 biomolecular imager (GE Healthcare Bio-Sciences AB, Uppsala, Sweden) (excitation at 488 nm and emission at 530 nm), and binding to different PBPs was determined from at least three independent experiments using ImageQuant TL software v8.1.0.0 (GE Healthcare Bio-Sciences AB, Uppsala, Sweden).

In parallel to the aforementioned assay, we performed analogous experiments in which isolated PBP-containing membrane preparations instead of intact cells were pre-exposed to avibactam. Briefly, membrane preparations containing PBPs were obtained by following previously described protocols [8] and were incubated for 15, 30 and 60 min at 37 °C in the presence of 4 µg/mL avibactam and were afterwards labeled with bocillin FL (25 μM). Determination of the PBP fraction unbound was performed as described in the above section.

### 3.3. Determination of PBP-Binding Affinity (IC_50_)

PBP-binding affinity was determined using previously described methods [8]. Briefly, *P. aeruginosa* PAO1 cultures grown to the midexponential phase (7.6 log_10_ CFU/mL) were incubated in CAMHB at 37 °C and 180 rpm, washed and centrifuged (3220× *g* for 10 min at 4 °C). Bacterial pellets were washed in 30 mL of PBS buffer (pH 7.5), resuspended in 10 mL of PBS and sonicated at 40% amplitude for three 2 min bursts (while immersed in an ice bath) and centrifuged at 3220× *g* for 15 min at 4 °C. PBP-containing membranes were isolated by ultracentrifugation at 150,000× *g* for 30 min at 4 °C. Total protein concentrations were measured through the Bradford method.

To measure the 50% inhibitory concentrations (IC_50_), PBP-containing membrane preparations (0.5 mg/mL) were incubated at 37 °C for 30 min in the presence of increasing concentrations of avibactam, cefoxitin or imipenem (range of concentrations tested: 0.25–32 µg/mL). Subsequently, the fluorescent penicillin bocillin FL was added to a 25 μM final concentration [32]. Labeled PBPs were denatured and separated through 4–15% SDS–polyacrylamide gel electrophoresis. Labeled PBPs were visualized using a Typhoon FLA 9500 biomolecular imager (GE Healthcare Bio-Sciences AB, Uppsala, Sweden) (excitation at 488 nm and emission at 530 nm), and binding was determined from at least three independent experiments using ImageQuant TL software v8.1.0.0 (GE Healthcare Bio-Sciences AB, Uppsala, Sweden).

### 3.4. qRT-PCR Quantification of AmpC Induction

The induction of AmpC production by imipenem, cefoxitin and avibactam was determined by measuring the *ampC* mRNA levels by quantitative real-time reverse transcription-PCR (qRT-PCR) in wild-type PAO1, PAΔ*dacB* (PBP4), PAΔ*dacBdacCpbpG* (PBP4, 5/6 and 7), 101-E5 and 109-E9 (OprD Q142X, AmpR G154R) TOL/TAZ-sensitive clinical isolates and their respective isogenic resistant clinical isolates 103-H8 (AmpC E247K; PDC-221) and 110-G8 (AmpC DelG229–E247; PDC-223). To this end, late exponential-growing *P. aeruginosa* cultures of PAO1, PAΔ*dacB*, PAΔ*dacBdacCpbpG*, 101-E5, 109-E9, 103-H8 and 110-G8 (dilution: 1/100) were preincubated in CAMHB at 37 °C (180 rpm) for 20 min. Afterwards, imipenem (IPM; 8 µg/mL), cefoxitin (FOX; 64 µg/mL) or avibactam (AVI; 16 µg/mL) were added, and bacterial cultures were incubated at 37 °C, 180 rpm. Samples were taken at 0, 30 and 180 min. Total RNA was extracted with the RNeasy Mini Kit (Qiagen, Hilden, Germany) and treated with TURBO DNase (Thermo Fisher Scientific, Waltham, MA, USA) to remove contaminating DNA. Samples were afterwards normalized to 50 ng/µL.

Normalized samples were then used for one-step reverse transcription and real-time PCR amplification using the QuantiTect SYBR green qRT-PCR kit (Qiagen, Hilden, Germany) in a CFX Connect Real-Time PCR System (Bio-Rad, Hercules, California, USA). Previously described primer pairs ACrnaF/ACrnaR and rpsL-1/rpsL-2 were used for the amplification of *ampC* and housekeeping gene *rpsL*, respectively [3]. The mean values of relative mRNA expression (2^−ΔΔC_t_^) [33] obtained from at least two independent biological replicates and duplicate technical replicates were considered.

## Figures and Tables

**Table 1:** PBP-binding affinities (IC_50_) of imipenem, cefoxitin and avibactam for *P. aeruginosa* PAO1 isolated PBP-containing membranes.

*P. aeruginosa* PAO1 PBP *^a^*	Mean IC_50_ ± SD (µg/mL) *^b^*		
	Imipenem	Cefoxitin	Avibactam
1a	0.2 ± 0.06	7.8 ± 0.5	>32
1b	0.1 ± 0.03	>32	3.6 ± 0.1
2	0.05 ± 0.02	14.6 ± 2.2	4.2 ± 1.6
3	0.3 ± 0.07	10.7 ± 1.7	>32
4	0.1 ± 0.04	1.5 ± 0.5	3.1 ± 1.3
5/6	0.5 ± 0.2	9 ± 2.4	2.2 ± 0.7

*P. aeruginosa* PAO1 cultures were grown to the midexponential phase (7.6 log_10_ CFU/mL), and PBP-containing membranes were isolated by ultracentrifugation. Growing concentrations (range: 0.25–32 µg/mL) of the indicated compounds were added to the membrane preparations (0.5 mg/mL) during the 30 min binding reaction before labeling with 25 µM bocillin FL. Labeled PBPs were separated by SDS-PAGE and detected using a Fluorimager. *^a^* PBP, penicillin-binding protein. *^b^* Mean values ± standard deviations from at least 2 independent experiments are shown.

**Table 2:** Basal *ampC* qRT-PCR expression for the studied *P. aeruginosa* strains.

*P. aeruginosa* Strain *^a^*	*ampC* Expression (min) *^b^*		
	0	30	180
PAO1	1	2.6 ± 0.5	5.8 ± 0.1
PAΔ*dacB*	110 ± 0.8	228 ± 117	1100 ± 243
PAΔ*dacBdacCpbpG*	7500 ± 3800	27,000 ± 9400	58,000 ± 26,000
101-E5	554 ± 273	466 ± 272	2400 ± 1100
103-H8	391 ± 345	317 ± 21.2	5400 ± 2900
109-E9	414 ± 122	496 ± 213	1700 ± 382
110-G8	306 ± 31.2	418 ± 183	3500 ± 1600

*^a^* The strains studied are: wild-type PAO1, PAΔ*dacB* (PBP4 knockout mutant), PAΔ*dacBdacCpbpG* (PBP4, 5/6 and 7 triple knockout mutant) 101-E5 and 109-E9 (OprD Q142X, AmpR G154R) CAZ/AVI-sensitive ST175 high-risk clone clinical isolates and 103-H8 (AmpC E247K; PDC-221) and 110-G8 (AmpC DelG229–E247; PDC-223) CAZ/AVI-resistant ST175 high-risk clone clinical isolates. *^b^* Relative *ampC* mRNA expression (with respect to wild-type PAO1) without induction (basal) was assessed via qRT-PCR. The mean values from at least two sets (biological replicates) of two technical replicates ± standard deviation are shown.

## Data Availability

The data presented in this study are openly available in Mendeley Data at doi: 10.17632/45x4wyf96f.1 (http://dx.doi.org/10.17632/45x4wyf96f.1).
